# The Bi-Functional Organization of Human Basement Membranes

**DOI:** 10.1371/journal.pone.0067660

**Published:** 2013-07-03

**Authors:** Willi Halfter, Christophe Monnier, David Müller, Philipp Oertle, Guy Uechi, Manimalha Balasubramani, Farhad Safi, Roderick Lim, Marko Loparic, Paul Bernhard Henrich

**Affiliations:** 1 Department of Neurobiology, University of Pittsburgh, Pittsburgh, Pennsylvania, United States of America; 2 Biocenter of the University of Basel, Basel, Switzerland; 3 Proteomics Core Facility, University of Pittsburgh, Pittsburgh, Pennsylvania, United States of America; 4 Ophthalmology Department, University of Pittsburgh, Pittsburgh, Pennsylvania, United States of America; 5 Ophthalmology Department of the University Basel, Basel, Switzerland; Emory University School of Medicine, United States of America

## Abstract

The current basement membrane (BM) model proposes a single-layered extracellular matrix (ECM) sheet that is predominantly composed of laminins, collagen IVs and proteoglycans. The present data show that BM proteins and their domains are asymmetrically organized providing human BMs with side-specific properties: A) isolated human BMs roll up in a side-specific pattern, with the epithelial side facing outward and the stromal side inward. The rolling is independent of the curvature of the tissue from which the BMs were isolated. B) The epithelial side of BMs is twice as stiff as the stromal side, and C) epithelial cells adhere to the epithelial side of BMs only. Side-selective cell adhesion was also confirmed for BMs from mice and from chick embryos. We propose that the bi-functional organization of BMs is an inherent property of BMs and helps build the basic tissue architecture of metazoans with alternating epithelial and connective tissue layers.

## Introduction

Basement membranes (BMs) are thin sheets of extracellular matrix (ECM) at the basal side of every epithelium. They outline muscle fibers and are present at the basal surface of the vascular endothelial cells [1]. Despite their presence in all organs of the body, BMs are difficult to isolate, and the identification of typical BM constituents was only possible after realizing that yolk sac tumors produce large quantities of a BM-like ECM [2], [3]. BM proteins are typically multi-domain proteins of high molecular weights that either polymerize and (or) bind to other BM proteins and consequently form thin extracellular matrix sheets. The binding of BM proteins to cellular receptors, such as integrin family members [4], [5] and dystroglycan [6] is required for BM assembly. The importance of BMs for metazoans is evident by their evolutionary conservation and the dramatic phenotypes from worms to humans with mutations that affect the assembly or stability of BMs [7]–[17].

The current model states that thin BMs are one-layered extracellular matrix sheets that are composed of a two-dimensional network of collagen IV connected to a network of polymerized laminin [1]. Nidogen-1 has been proposed to provide the connection between the two polymers [18]. The model was designed based on in-vitro-binding assays and electron microscopy imaging of purified collagen IV and laminin 111 that spontaneously form network-like structures [19].

The majority of BMs have a thickness of less than 100 nm, and a major constraint in designing a BM model is to fit the very large ECM proteins into the thin BM sheets. For example, the 100 nm thickness of BMs allows the up to 400 nm-long long collagen IVs to be positioned only in a horizontal configuration. However, thickness measurements of BMs have been traditionally based on transmission electron microscopy (TEM) imaging that requires chemical fixation and dehydration of the samples. Recently, the thickness of BMs was re-examined using atomic force microscopy (AFM) and confocal microscopy [20]–[23]; both techniques allow studying fully hydrated and unfixed BMs. The AFM studies on the chick ILM and human ILMs, as well as confocal microscopy studies on the lens capsule showed that the native and hydrated BMs are between two and four-times thicker than previously recorded. The AFM studies also showed that a major part of BMs is water that is tightly bound by the GAG side chains of proteoglycans [20]–[22]. With a much greater thickness, a non-horizontal positioning of ECM proteins in BM sheets would also be possible.

Due to the difficulty of handling the very delicate in vivo-derived BMs and the lack of suitable experimental methods, most data on BM structure and function are based on binding assays with recombinant BM proteins and the phenotype analyses of mice, worms and zebra fish with mutations of BM proteins. The present data show that BMs from longer-living species, such as humans, are thicker and, therefore, easier to test for side-specific properties. The present experimental data with three different human BMs provides new insights into the general biology of in vivo-derived BMs and allows proposing a new concept how BMs are functionally structured.

## Methods

### Basement Membranes

Human cadaver eyes (n = 20) ranging from 19 to 84 years of age were obtained from CORE, the Center of Organ Recovery and Education (Table S1). The eyes were harvested within 4 to 24 hours of death and delivered to the laboratory in less than two days. The use of the human eyes for this project was approved by the internal review board of the University of Pittsburgh under the IRB protocol number # 0312072. The inner limiting membrane (ILM) was obtained by incubating segments of retinas in 2% Triton-X-100 in dest. water overnight, and transferring the ILMs in new detergent that also included 2% deoxycholate [20]. The Descemet’s membrane (DM) was obtained from corneas that were incubated in 2% Triton-X-100 for 3 hours followed by micro-dissection of the DM from the corneas. The lens capsule (LC) was obtained by dissection alone. Flat mounting of the BMs was achieved by suspending segments of DM, LC or ILM in a droplet of PBS on poly-lysine-coated slides, draining the liquid and firmly immobilizing the BMs by centrifugation at 1200 rpm for 5 minutes. Neonatal and adult mouse and embryonic chick ILMs were isolated by either detergent treatment of the retinas [24] or by splitting of flat-mounted retinas followed by detergent treatment [25]. ILM samples were also obtained from surgical ILM peeling to treat macular holes (n = 6; IRB # PRO12120223).

### Immunocytochemistry

The flat mounted BMs were incubated with primary antibody for 3 hours to overnight, washed, and incubated with Cy3, Cy2 or peroxidase-labeled secondary antibodies (Jackson ImmunoResearch, West grove, PA) for 2 hours. For staining of cross sections, the samples were fixed in 4% paraformaldehyde, cryoprotected in 25% sucrose, embedded in OCT compound and sectioned at 25 µm. The sections were mounted on poly-lysine-coated Superfrost Plus slides (Fisher Scientific, Pittsburgh, PA). Some ILM preparations were whole-mount-labeled for laminin, embedded in Technovit 7100 (EMS, Fort Washington, PA), and sectioned at 3 µm. The plastic-embedded sections were then stained for collagen IV 7S (see below). Images were taken with an Olympus FlowView confocal microscope. For TEM, the labeled BMs were reacted with DAB, post-fixed and embedded in EPON according to standard protocols. Two polyclonal antibodies to mouse laminin-111 (Sigma, St.Louis, MO; Invitrogen, Carlsbad, CA) and a monoclonal antibody to human laminin α5 (Invitrogen) were used to detect laminin in the human BMs. Two rabbit polyclonal antibodies (Rockland, Gilbertsville, PA; ICN/Cappel, Aurora, OH) to human placental collagen IV were used to localize collagen IV protein in BMs. Rat monoclonal antibodies to C-terminal domains of all 6 collagen IV α chains were provided by Dr. Y. Sado. The antibodies were raised to unique, chain-specific NC1-peptides [26]. A 7S-specific mouse monoclonal antibody (Mab J3-2) was provided by Dr. Nirmal SundarRaj. The Mab is also available from Sigma. It was raised against human placental collagen IV, and it was ELISA-tested against placental collagen IV [27] and the 7S domain of human placental collagen IV (pers. communication Dr. SundarRaj). The specificity of the antibody was further investigated by western blotting and immunoprecipitation (see Results).

For immunoprecipitation, human lens capsules were each digested in 100 µl of 100 U/ml collagenase in PBS (Type VII; Sigma) overnight at 37°C. After centrifugation, the solubilized ECM proteins were incubated with 5 µl of Mab J3-2 ascites or anti-laminin antiserum and the antibody-bound peptides were immunoprecipitated with 50 µl of anti-mouse IgM-Sepharose (Sigma). Following centrifugation and three-times washing, the beads were boiled in 8 M urea/SDS sample buffer, and the released peptides were separated by SDS PAGE and western blotted. The blots were incubated with a polyclonal anti-collagen IV antibody (Rockland) followed by alkaline-phosphatase-conjugated goat anti-rabbit (1∶2000; Jackson ImmunoResearch, West grove, PA). The proteins were detected by reaction with NBT/BCIP (Roche).

### Atomic Force Microscopy (AFM) Testing of the Bms

The stiffness of the epithelial or stromal side of BMs was determined by “forced indentation” using a Nanowizard I Atomic Force Microscope (JPK Instruments, Berlin, Germany) and a FlexAFM “ARTIDIS” (Nanosurf AG, Liestal, Switzerland), which were mounted on a Zeiss Axiovert 135 TV and Zeiss Axiobserver A1 inverted microscopes (Zeiss AG, Oberkochen, Germany) respectively. The experimental details were described previously [28].

### Cell Adhesion Assays

Single cell suspensions of MDCK cells (American Type Culture Collection, ATCC; kindly provided by Dr. Luciana Gallo), rabbit corneal epithelial (kindly provided by Dr. Nirmala SundarRaj and Katarine Davoli; [29]), human dermal endothelial cells (ATCC), embryonic chick retinal cells and human fibroblasts were suspended at 200 000 cells/ml in DMEM/2% ovalbumin and plated onto the flat mounted BMs. After incubation for 15 minutes, the non-adherent cells were washed off with PBS and the preparations were fixed in 4% paraformaldehyde, 0.05% glutaraldehyde for 15 minutes. The adherent cells were detected by nuclear dye staining using Sytox Green or Sytox Orange (Molecular Probes/Invitrogen). Adherent cells were counted per unit area of 300×200 µm on the epithelial and (or) stromal side of the same BM with three counts per preparation and with an “n” of at least 3 biological repeats. The average was calculated with standard deviation, and the statistical significance was confirmed by Students T-tests. Dorsal root ganglion and retinal explants from E6 chick embryos were prepared as described [30]. Axon outgrowth was detected by staining the cultures with a monoclonal antibody to tubulin (mAb 6G7; Developmental Studies Hybridoma Bank, University of Iowa) after 24 hours of incubation. Some of the ILMs were treated with 0.15 µU/µl chondroitinase ABC (Seikagaku) or 1 mg/ml hyaluronidase (Sigma) in PBS/BSA for 6 hours prior to the MDCK adhesion outgrowth assays (n = 5 for each assay).

## Results

**Figure 1:**
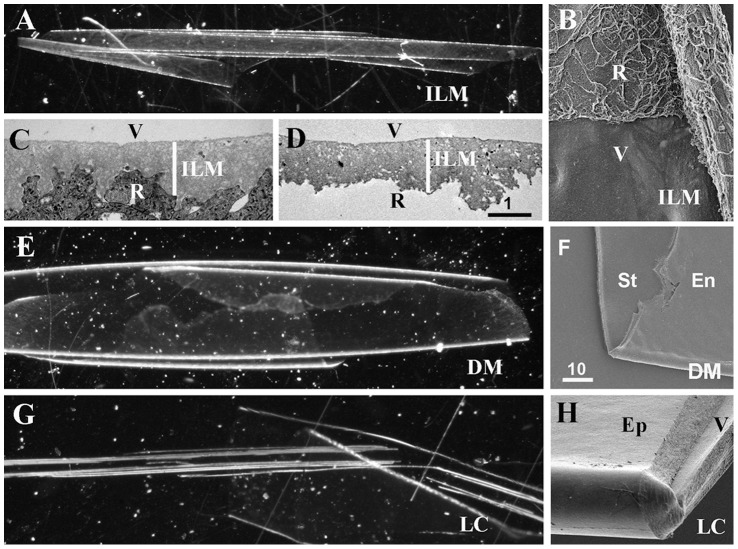
Isolated human ILM (A), Descemet’s membrane (DM, E) and lens capsule (LC, G) as they appear under a dissecting microscope using dark field. All BMs are transparent and rolled up, whereby the epithelial surfaces are facing the exterior and the stromal surfaces is facing the interior of the rolled-up BM sheets. SEM micrographs of a folded ILM (B), a DM (F) and a LC (H) show the two surfaces of the BMs. As shown in panel (B), the irregular retinal (R) surface of the ILM can be readily distinguished from the smooth vitreal surface (V), consistent with the morphological differences seen in TEM micrographs of crossections of ILMs in situ (C) and isolated ILMs (D). The endothelial (En) surface of the DM and epidermal (Ep) surface of the LC are indistinguishable from their stromal or vitreal (V) surfaces (F, H). Scale bars: C, D: 1 µm; B, F; H: 10 µm.

Three types of basement membranes were isolated from 19 to 84-old human donor eyes: the inner limiting membrane (ILM), a thin BM that separates the retina from the vitreous; the lens capsule (LC), a thick BM that envelopes the lens epithelium and lens fibers, and Descemet’s membrane (DM), a BM of intermediate thickness that is located between the corneal endothelium and the corneal stroma. Common to all of these BMs is that they have an epithelial side that, in vivo, is apposed to either the retinal, the lens epithelial or the corneal endothelial cell layers, and a stromal side that is adjacent to the a-cellular vitreous (ILM, LC) or the collagen-rich corneal stroma (DM). Regardless of age, all BMs, when isolated, were transparent and rolled up, whereby their epithelial sides were consistently facing outward, whereas the stromal sides were facing inward (Fig. 1A, E, G). In the case of the ILM, the rolling is according to the native curvature of the retina, but in the case of the DM and LC, the rolling direction is opposite to the native curvature of the cornea and lens. The epithelial and the stromal surfaces of the ILM were morphologically distinct, consistent with the morphological differences seen in situ (Fig. 1B–D). The two sides of the LC and DM appeared morphologically similar (Fig. 1F, H). Thanks to the consistent rolling, the isolated BMs could be predictably flat-mounted with either the epithelial or the stromal side facing up for biophysical testing, immunocytochemistry and cell adhesion assays. In an alternative technique, the BMs were flat-mounted in a folded configuration whereby the epithelial and stromal side became exposed side-by-side.

### Side-specific Surface Properties of BMs

**Figure 2:**
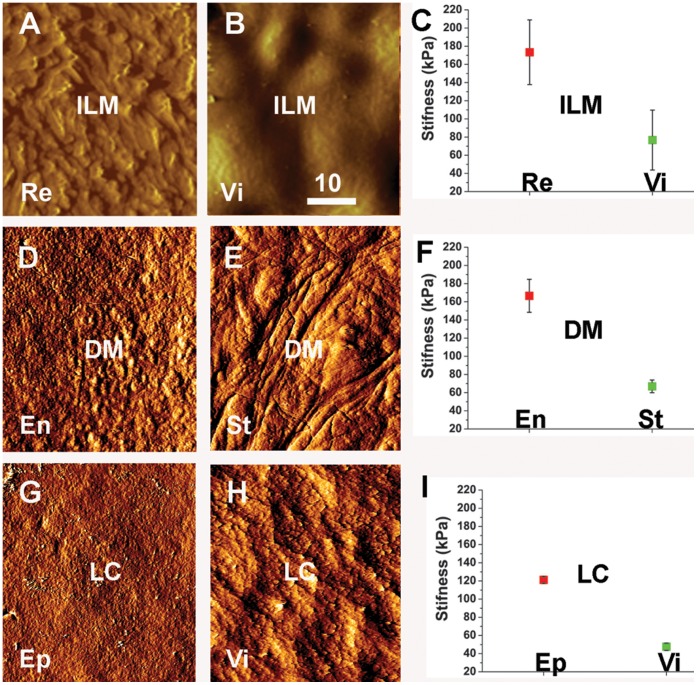
AFM testing of the two surfaces of the ILM (A–C), the DM (D–F) and LC (G–I). The AFM imaging mode shows the morphological differences between the retinal (Re)/epithelial (Ep)/endothelial (En) surfaces and the vitreal (Vi)/stromal (St) surfaces of the ILM, DM and the LC. The graphs in (C, F, I) show the quantification of the stiffness measurements obtained by AFM “forced indentation”. The measurements were obtained by probing five ILMs, three DMs and three LCs. The epithelial surfaces of all tested BMs were about twice stiffer than the stromal surfaces. The differences were statistically significant. Scale bar: 10 µm.

The surface structure and the biomechanical properties of flat-mounted BMs were studied using atomic force microscopy (AFM). With the imaging mode of the AFM, it was found that the surface structure of the vitreal and retinal side of the ILM were different, whereby the vitreal surface was even and smooth and the epithelial side highly irregular with numerous indentations and peaks (Fig. 2A, B). The morphological differences seen by AFM are consistent with the differences seen in SEM images of isolated ILMs (Fig. 1B) and TEM images of ILMs in situ (Fig. 1C). The morphological differences of the epithelial and the stromal surfaces of the DM (Fig. 2D, E) and the LC (Fig. 2G, H) were not as obvious as for the ILM. In the “forced indentation mode”, AFM probing of the ILMs DMs and LCs revealed that the stiffness of the epithelial sides of all tested BMs was approximately 50% higher than the stiffness of the stromal sides (Fig. 2C, F, I).

Previous experiments with human and chick ILMs had shown that the enzymatic removal of the GAG side chain of proteoglycans resulted in an increase in the stiffness of the BM due to loss of water that is tightly bound by the GAG chains in the BMs [20]. We expected a similar increase in stiffness due to water loss when ILM samples were probed in the presence of high molar salt (2 M sodium chloride). To test this, the retinal and the vitreal side of ILM samples were AFM-tested at isotonic, hypotonic and hypertonic salt concentrations. While the stiffness of the vitreal side of the ILMs remained the same at all salt concentrations (Fig. S1A; n = 3), the stiffness of the retinal side was doubled in high salt as compared to isotonic or hypotonic buffers (Fig. S1B, C; n = 3), establishing an additional distinguishing property between the two sides of this BM. The result also indicated that the retinal side of the ILM has a greater water content than the vitreal side at physiological salt concentration.

### Side-specific Protein Distributions in BMs

**Figure 3:**
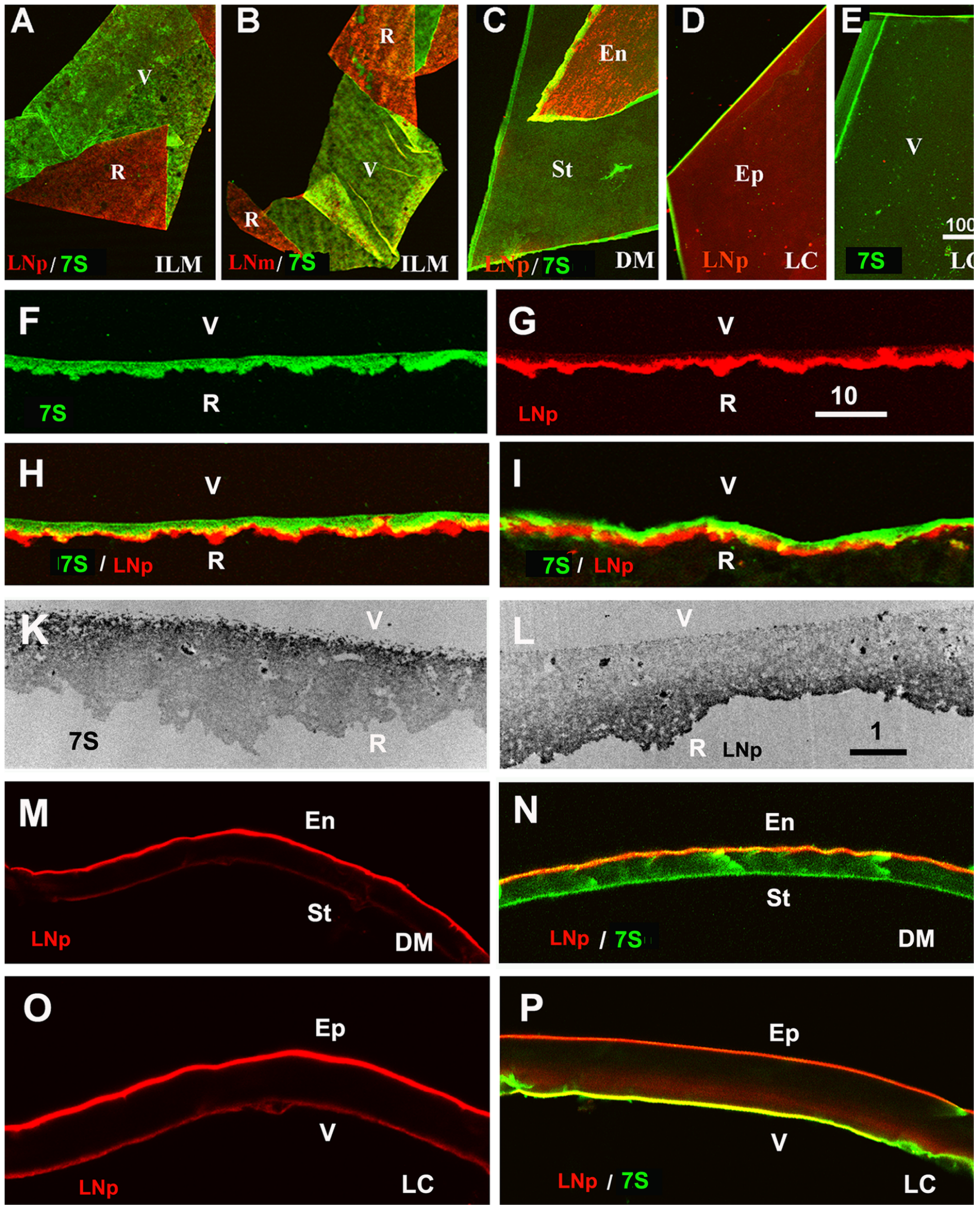
The asymmetric structure of human BMs as shown by double labeling of folded ILMs (A, B), folded Descemet’s membranes (DM, C) and differently mounted lens capsule segments (LC, D, E). The retinal surface (R) of the ILM and the endothelial (En) and epithelial (Ep) sides of DM and LC were stained with a polyclonal (red, LNp; A, C, D) and a monoclonal antibody to laminin (red, LNm, B), whereas the vitreal (V) or stromal side (St) of the BMs were labeled with an antibody to the 7S domain of collagen IV α3/4/5 (green; A-C, E). The asymmetry of BMs was also detected by single and double labeling of crossections of an isolated ILM (F-H) and an ILM in situ (I). The TEM micrographs in panel (K, L) show crossections of isolated ILMs stained for 7S collagen IV α3/4/5 (K) and laminin (L). The dark label shows the localization of 7S collagen IV on the vitreal side (K) and laminin on retinal side of the ILM (L). An asymmetric distribution for laminin and collagen 7S was also detected for the Descemet’s membranes (M, N) and the lens capsule (O, P). The sections were stained for laminin (red; M-P) and collagen IV 7S (green; N, P). Scale Bars: A-E: 100 µm; F-I and M-P: 10 µm; K, L, I: 1 µm.

Immunocytochemistry of flat-mounted ILMs, DMs and LCs showed that laminin is prominently localized at the epithelial sides of all BMs (Fig. 3A–D). The asymmetric distribution of laminin was also detected in crossections of isolated ILMs (Fig. 3G, H), ILMs in situ (Fig. 3I, 7E), and crossections of isolated DM and LC (Fig. 3M–P). The abundance of laminin on the epithelial side of BMs was detected using two polyclonal anti-laminin antisera (Fig. 3A, C, D) and a monoclonal antibody that is directed to human laminin α5 (Fig. 3B). Laminin α5 is the most abundant laminin α chain in adult human BMs as found by immunocytochemsitry [31] and proteomic analysis [32].

Collagen IV is the most abundant protein in adult human BMs. The hetero-trimeric protein is comprised of a central, triple helical collagenous domain, a non-collagenous “NC1 domain” at its C-terminus, and an N-terminal non-collagenous “7S domain”. The NC1 domain of a collagen IV associates with the NC1 domain of another collagen IV to form a dimeric supra-structure, whereas the 7S domain associates with the 7S domains of three other collagen IVs to form a tetrameric association. The 6 collagen IV alpha chains assemble into 3 distinct collagen IV trimers. To localize all three collagen IV members and all of their sub-domains in BMs, we used two polyclonal anti-collagen IV antisera. In addition, we used monoclonal antibodies that are specific to the NC1 domains of all 6 of collagen IV alpha chains.

**Figure 4:**
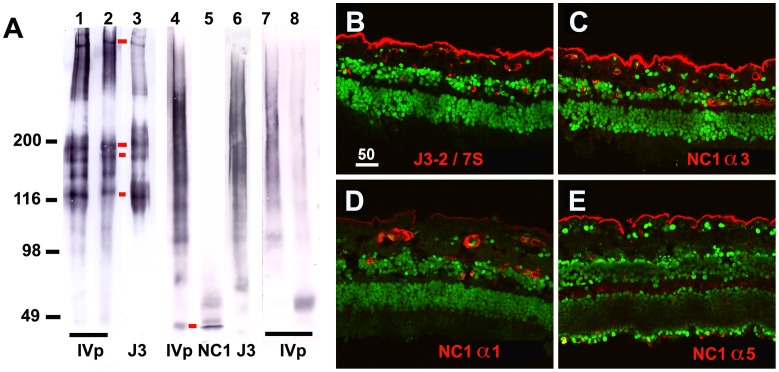
Domain andα chain-specificity of Mab J3-2, a monoclonal antibody that recognizes the 7S domain of α3/4/α5 collagen IV. The domain specificity was determined by Western blotting (lanes 1–6, panel A) and immunoprecipitation (lanes 7, 8, panel A) using adult human lens capsules as sample. The western blot of lanes 1 and 2, stained with a polyclonal antiserum to human collagen IV, shows the complex peptide banding pattern of intact collagen IV from human lens capsules age 24 (lane 1) and age 65 (lane 2). Staining of the 65 year-old lens capsule sample with the J3-2 Mab (lane 3) shows a similar, but not entirely identical banding pattern. Red bars mark corresponding bands. The non-collagenous (NC1 and 7S) domains of collagen IV were detected in western blots after digestion of lens capsules with collagenase: western blots with the soluble supernatant from digested lens capsule samples, stained with polyclonal collagen IV antiserum, shows the NC-domains of collagen IV as a long smear at 300 kD and a ladder of lower molecular weight peptides (lane 4, panel A). The NC1 domain of α3 collagen IV appears as a sharp band below 49 kD (lane 5, red bar; panel A) as shown by staining with a monoclonal antibody specific to the collagen IV α3 NC1 domain. The high molecular weight smear stained by Mab J3-2 correspond to the variably crosslinked 7S domain (Lane 6, panel A). The smear is due to the glycosylation of the 7S domain [60], [61]. For immunoprecipitation, the collagenase-digest of lens capsule was incubated with Mab J3-2 or anti-laminin as a control followed by anti-mouse IgM or anti-rabbit IgG agarose. The beads were washed and the bound peptide released by boiling in high molar urea/SDS sample buffer. The released peptides were separated by SDS PAGE and western blotted. The immuno-precipitated peptides were visualized in the blot by a polyclonal anti-collagen IV antibody/alkaline phosphatase-conjugated secondary antibody. For the J3-2 pull-down, a smear of approximately 300 kD was detected (lane 7, panel A), similar to the smear detected by Western blotting (lane 6). A control pull-down with anti-laminin is shown in lane 8. The blots shows that Mab J3-2 i) recognizes human collagen IV, that it ii) recognizes an NC domain of collagen IV and that iii) the molecular weight of the detected peptides are different from the molecular weight of the NC1 domain but identical to the molecular weight of the 7S domain of collagen IV from glomerular BM and lens capsule. To determine the chain-specificity of the antibody, sections of adult human retina were stained with Mab J3-2 (B), to the NC1 domain of collagen IV α3 (C), to the NC1 domain of collagen IV α1 (D) and to the NC1 domain of collagen α5 (E). A3, α4 (not shown) and α5 collagen IV are very prominent in the ILM but sparse in the BMs of the retinal blood vessels (C, E), whereas α1/2 was very sparse in the ILM but abundant in the blood vessels (D). The staining with Mab J3-2 (A) resembles most closely the staining of collagen α3 α4 and α5, and it is very different from the distribution of collagen IV α1/α2. Scale Bar: 50 µm.

An antibody directed to the 7S domain was used for this project as well (Mab J3-2) [27]. Experiments showed that this antibody stained preferentially the vitreal/stromal side of all tested BMs and, therefore, served as a marker for the vitreal/stromal surface of human BMs. Because of the importance of this antibody for this project, western blotting and immunoprecipitation was employed to confirm the domain and alpha chain-specificity of this antibody: Western blots with samples of intact human lens capsules and stained with Mab J3-2 showed a series of diffuse bands that are in part identical to the bands labeled with a polyclonal anti-collagen IV antibody (4A, lanes 1-3). With samples of soluble supernatant from human lens capsule that were digested with collagenase, the J3-2 Mab labeled a series of diffuse bands (Fig. 4A, lane 6) that were also in part identical with the bands detected with polyclonal anti-collagen IV antiserum (Fig. 4A, lane 4), but were different to the low molecular weight band detected with a monoclonal antibody to the NC1 domain of collagen IV α3 (Fig. 4A, lane 5).

For immunoprecipitation, human lens capsules were each digested with collagenase and the non-collagenouse peptides precipitated with J3-2 or laminin. The precipitated proteins were detected in western blots by staining with polyclonal antiserum to collagen IV. The J3-2-precipitate ran as a smear of app. 300 kD (Fig. 4A, lane 7). The high molecular weight smear of the J3-2 precipitate corresponds to the glycosylated and cross-linked terameric collagen IV 7S, consisting of a total of 12 7S peptides of each 26 kD. The banding pattern is identical with the banding pattern of the tetrameric 7S domain of collagen IV isolated from human glomerular BM and lens capsule [33]. It is of note that the 7S domain from EHS mouse tumor has a different protein-banding pattern with a peptide ladder from 153 to 27 kD (compare with Fig. 2b lane 1, 4 in Risteli et al. [34]). This is most likely due to lesser crosslinking. The control precipitation with anti-laminin is shown in Fig. 4A, lane 8.

**Figure 5:**
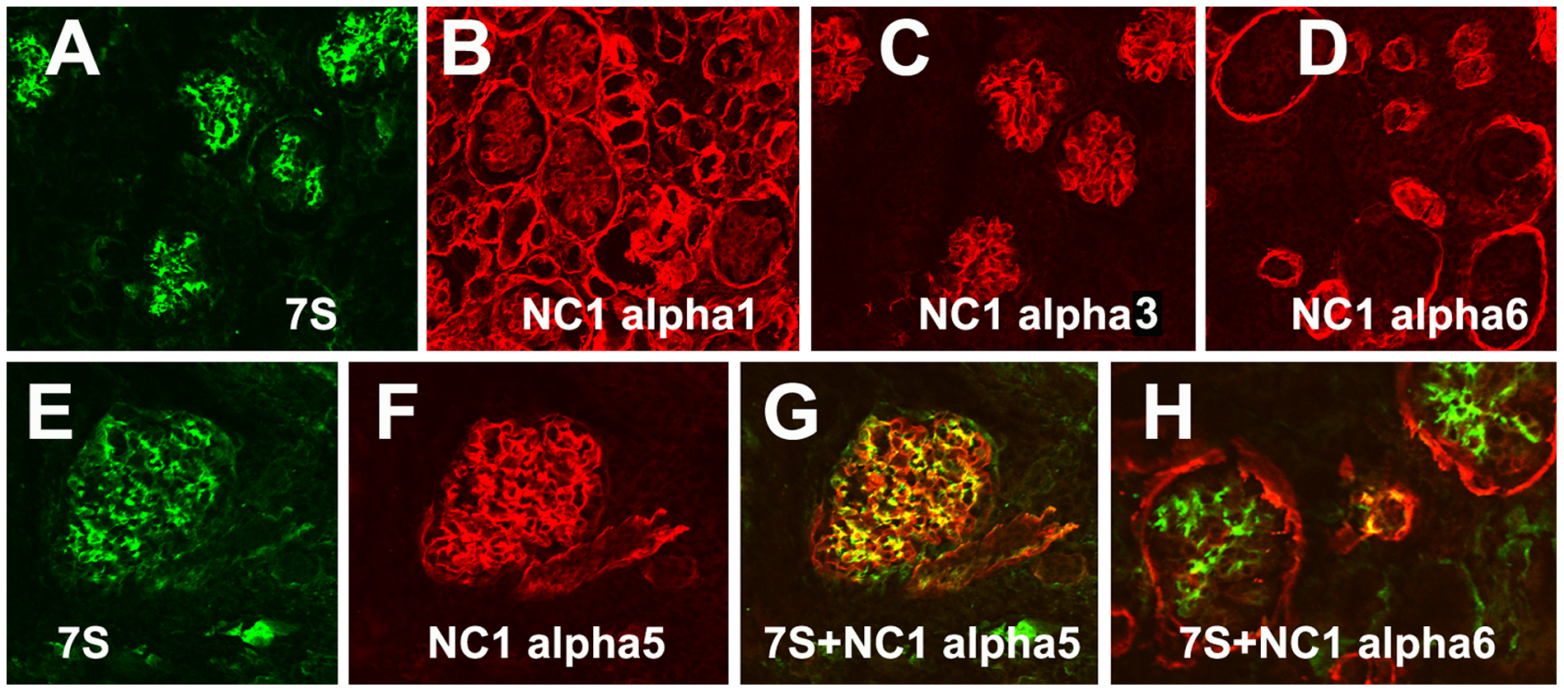
Collagen IV distribution in the embryonic human kidney. Sections of 20-week fetal human kidney were stained with 7S-specfic Mab J3-2 (7S, A, E) and antibodies to the NC1 domain of collagen IV α1 (B), to the NC1 domain of collagen IV α3 (C), to the NC1 domain of collagen IV α5 (F) and to the NC1 domain of collagen IV α6 (D). A3/4/5 collagen IV is very prominent in the glomerular BMs (C, F), whereas α1/2 was equally abundant in all BMs of the kidney (B). The α6 chain is present in the BM of Bowman’s capsule (D). The staining with Mab J3-2 (7S, A, E) resembles most closely the staining for collagen α3 (C) and α5 (F), and it is different from the distribution of collagen IV α1/2 (B) and α6 (D). Double labeling with Mab J3-2 and the anti-NC1 α5 (G) or the α6 (H) shows the overlap with of 7S with α5 staining and the different distribution with α6 (H). Bar: 50 µm.

The collagen IV α-chain specificity of Mab J3-2 was established by immunocytochemsitry: the J3-2 Mab showed i) a very strong labeling of the ILM but faint staining of retinal blood vessels (Fig. 4B), ii) a preferential staining of the glomerular BMs of the human kidney (Fig. 5A, E) and iii) no staining of the epidermal BM of human skin (not shown). When compared with the labeling patterns obtained with all six NC1-chain-specific collagen IV antibodies, the staining pattern of Mab J3-2 was very similar to that of α3, α4 and α5 collagen IV in the retina (Fig. 4C, E) and kidney (Fig. 5C, F) and different to the labeling for α1/2 and α6 collagen IV (Fig. 4D, Fig. 5B, D): α1/2 collagen IV is i) absent in the ILM but very prominent in the BMs of the retinal vasculature (Fig. 4D), ii) is uniformly present in all BMs in the kidney (Fig. 5B) and iii) very prominent in the epidermal BM of the skin (not shown). A6 collagen IV is absent in the ILM and prominent in the BM outlining Bowman’s capsule of the kidney (Fig. 5D [23]). Double labeling with Mab J3-2 and anti NC1 α3/4/5 or α6 showed an overlapping distribution with α3/4/5 (Fig. 5G) but a different distribution to α6 (Fig. 5H). The current data combined showed that Mab J3-2 recognizes the 7S domain of collagen IV α3/4/5.

The antibody staining data also showed that collagen IV α3/4/5 is the most prominent collagen IV in the human ILM (Fig. 4B, C, E), consistent with mass spectrometry data analyzing the proteome of the adult human ILMs [32].

The side-selective distribution of laminin and collagen IV 7S in ILM, DM and LC was best appreciated by double staining of whole mounts and cross sections (Fig. 3A–C; H, I, N, P). The side-selective abundance of 7S collagen IV and laminin in the ILM was also confirmed by TEM at high resolution (Fig. 3K, L). TEM also revealed that the highest concentration of laminin and collagen IV α3/4/5 is at each of the ILM’s top most surfaces.

**Figure 6:**
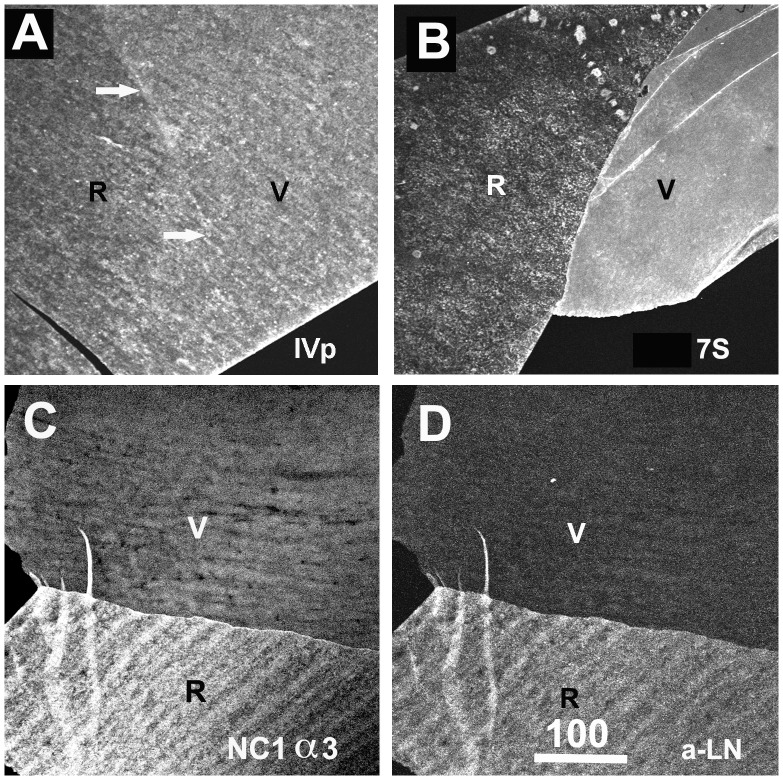
Detection of collagen IV in human ILM whole mounts. Staining with polyclonal antibodies resulted in a uniform and even labeling of the retinal (R) and vitreal (V) side of the ILM. In contrast, staining of ILM with antibodies specific to the 7S (B) or the NC1 domain (C) of collagen IV alpha3/4/5 resulted in the selective labeling of the vitreal (B) or the retinal side (C). The distribution of laminin in the ILM (D) is very similar to that of collagen IV NC1 at the epithelial side. The ILM sample in panel C and D was double-labeled; the NC1 domain was labeled with a Cy3 (red; C) secondary antibody, whereas laminin was detected with a Cy2 (green; D) secondary antibody. Scale Bar: 100 µm.

**Figure 7:**
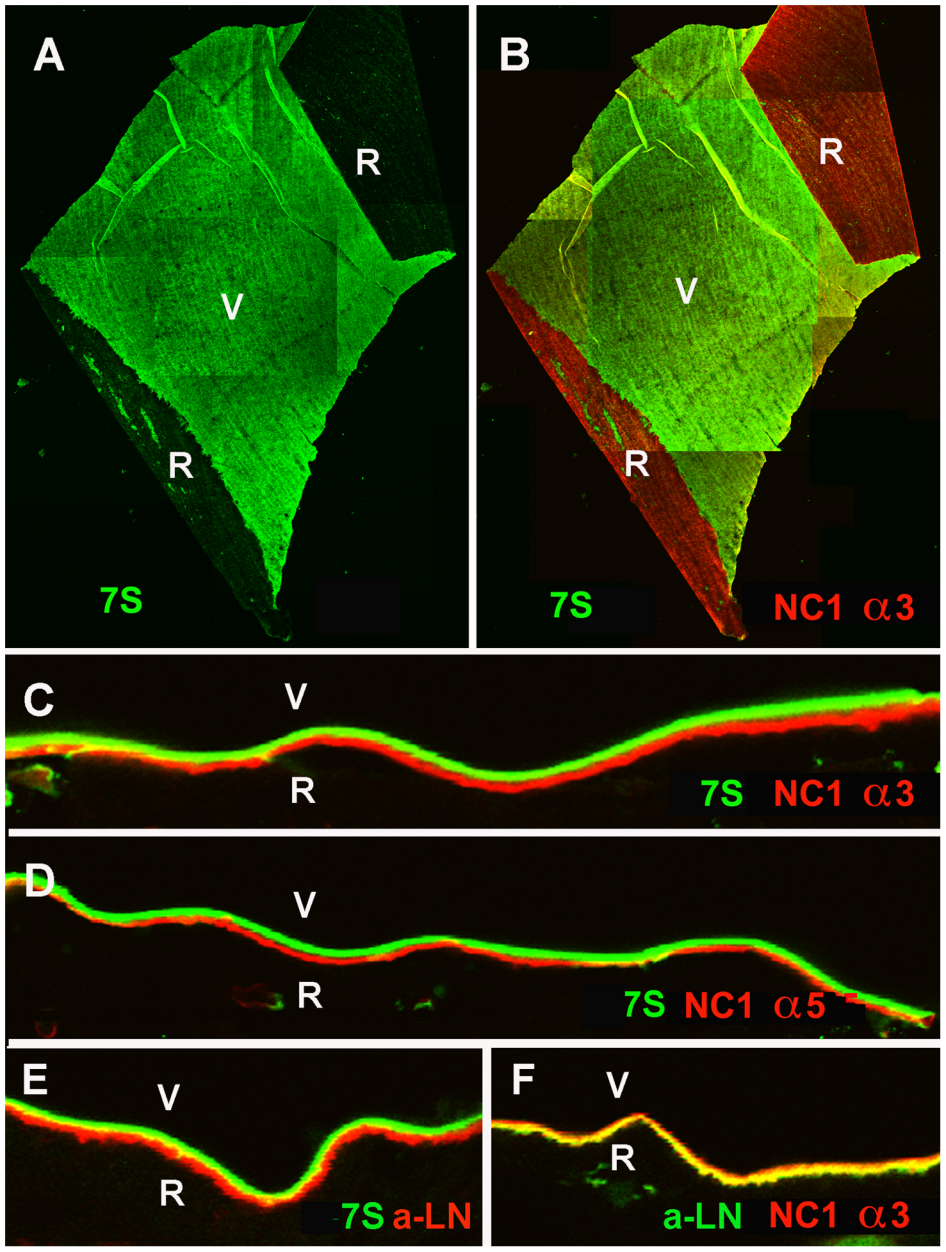
Distinct localization of the N and C-terminal domains of collagen IV in the human ILM. Labeling of ILM flat mounts with an antibody specific for the 7S domain of collagen IV α3/4/5 (7S) stained only the vitreal side (V) of the BM (green, A). The epithelial/retinal side of the ILM was prominently labeled with an antibody to NC1 domain of collagen IV α3 (B, red, NC1 α3) as shown by double labeling. Double labeling of retinal crossections with antibodies to the 7S domain of collagen IV α3/4/5 (green, C-E) and the NC1 domain of collagen IV α3 (NC1 α3, red, C) or α5 (NC1 α5, red, D) confirmed the distinct distribution of the C and N-terminal domains of collagen IV in this BM. Adjacent sections were stained for 7S of collagen IV α3/4/5 (green) and laminin (LN; red; E), and laminin (red) and NC-1 collagen IV α3 (green; F). Panel (E) shows the distinct localization of laminin and the 7S domain of collagen IV, and the yellow label in panel (F) the co-localization of laminin with the NC1 domain of collagen IV. Bar: A, B: 100 µm; C-F: 10 µm.

The prevalent hypothesis is that the collagen IVs use the NC1 and 7S binding sites to form a horizontally organized network that serves as the main scaffold for BMs [19]. According to this model, labeling of BMs with antibodies specific to the NC1 and 7S-domains should show a single-layered, overlapping distribution. This notion was tested by staining whole mounts and cross-sections of human BMs with polyclonal antisera that recognize all collagen IVs and all of their sub-domains, and with monoclonal antibodies specific to its 7S and NC1 domains. Staining of human ILM whole mounts with two polyclonal anti-collagen IV antibodies resulted in a uniform and even labeling of both sides of the BM (Fig. 6A). In contrast, labeling of ILM crossections or flat-mounts with the 7S-specific antibody resulted in a prominent labeling of only the vitreal side (Fig. 3F; 6B and Fig. 7A), whereas labeling for the NC1-domain of collagen IV α3/4/5 resulted in a prominent staining of the epithelial side of the ILM (Fig. 6C). The NC1 domain of collagen IV and laminin co-localized at the retinal surface of the ILM (Fig. 6D).

**Figure 8:**
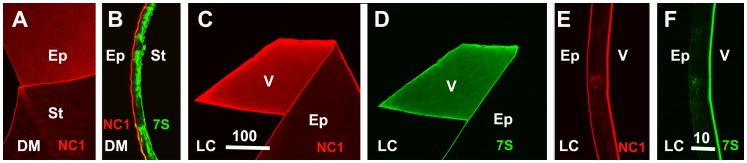
Staining of human DM and LC with domain-specific antibodies to collagen IV. Whole mounts and cross sections of DM (A, B) and LC (C-F) were stained for the NC1 domain (A, C, E) and (or) the 7S domain of collagen IV α3/4/5 (B, D, F). A labeled DM whole mount showed that the NC1 domain of collagen IV is prominently localized at the epithelia side of this BM (A). Double labeling of a DM crossection (B) showed a selective distribution of the NC1 domain at the epithelial side (red) and a prominent labeling for the 7S domain at the stromal side. Whole mounts (C, D) and crossections (E, F) of LC stained for the NC1 domain of collagen IV showed a strong labeling of the vitreal side and a weaker labeling of the epithelial side (C, E). The same whole mount and crossection stained for the 7S domain showed an almost exclusive labeling of the vitreal side (D, F). The data combined showed that the NC1 and 7S domain of collagen IV have a side-selective distribution in the DM, but do not have this side-selectivity in the LC. Scale Bar: A, C, D: 100 µm; B, E, F: 10 µm.

The side-selective distributions of the 7S and the NC1 domain of collagen α3/4/5 were confirmed by double staining of ILM whole mounts and crossections. While the 7S domain was almost exclusively localized to the vitreal side of the ILM (Fig. 7A), the NC1 domains of collagen IV α3, α4 and α5 were most prominent at the retinal side (Fig. 7B-D). Double labeling with antibodies to the NC1 domain of collagen IV and laminin showed that the C-terminal part of collagen IV and laminin co-localize at the epithelial side of the ILM (Fig. 7F). A similar epithelial versus stromal distribution of NC1 and 7S collagen IV α3/4/5 was detected for the DM (Fig. 8A, B). In LC, the NC1 domain was detected on both surfaces of this BM; the vitreal side of the LC, however, had a more prominent labeling (Fig. 8C, E), and in contrast to ILM and DM, the NC1 domain of collagen IV overlapped with the 7S domain at the vitreal side of this BM (Fig. 8D, F). It is of note that epitope unmasking by pre-treatments of the sections or BM whole mounts with 4 M urea or 0.1 M KCl pH1.5, as recommended for the immunocytochemical detection of collagen IV [26], had no effect on the staining patterns, but did enhance staining intensity. Staining of folded ILM flat-mounts with anti-fibronectin was performed as a control and showed no labeling (Fig. S2A, B). Fibronectin is not present in ILMs from non-diabetic patients.

An identical rolling and an identical side-specific distribution of laminin and collagen IV α3/4/5 was also detected for BM samples obtained by ILM peeling, the surgical removal of the BM from patient’s eyes (Fig. S3A–C). However, a consistent rolling pattern and a distinct protein sidedness was not detectable for mouse and embryonic chick ILMs using immunofluorescence staining (not shown).

### Side-specific Cell Adhesion to BMs

**Figure 9:**
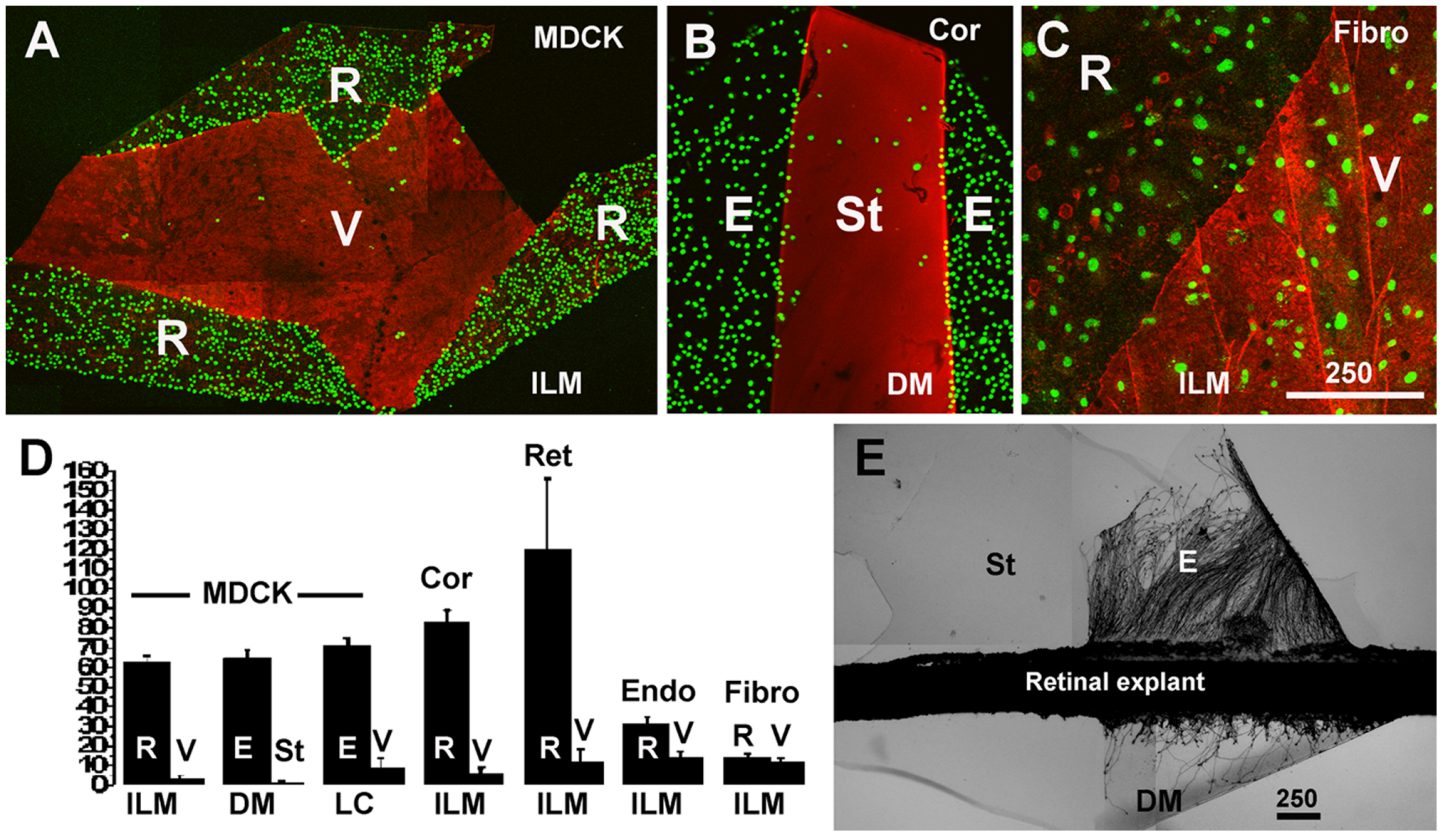
Cell adhesion assays revealing the sidedness of BMs. MDCK cells (A, B) and fibroblasts (C) were incubated on top of ILM (A, C) and DM (B). The BMs were folded to expose both the retinal/endothelial (R/E) and the vitreal/stromal (V/St) surface of the BMs. The non-adherent cells were washed off, and the firmly attached cells were visualized by nuclear dye staining (green). The BMs were stained for collagen IV 7S α3/4/5 (red) to visualize the V/St surface. The MDCK cells adhered almost exclusively to the R/E side of the folded BMs, and very few cells were detected on the 7S collagen IV-rich V/St side (A, B). Fibroblasts (Fibro) did not show this selective adhesion (C). Counting cells per unit BM surface area showed the strong preference of MDCK cells for the R/E side of the ILM, DM and LC. A similar adhesion preference was detected for corneal epithelial cells (Cor), embryonic chick retinal cells (Ret) and human vascular endothelial cells (Endo; p<0.001). Fibroblasts did not have a preference for either of the two BM sides. When a retinal explant strip was placed over a flat-mounted and folded DM (E), retinal axons only grew out on the endothelial (E) surface of the BM and not on the stromal surface (St). Scale bars: A-C, E: 250 µm.

Differences in the surface properties of the epithelial and the stromal sides of the human BMs were further tested in cell adhesion and neurite outgrowth assays (Fig. 9; Fig. S4). MDCK cells were the primary cell line used in the cell adhesion experiments. The kidney epithelium-derived cell line is regarded as the prototypic cell line to study classical epithelial characteristics, such as apico-basolateral polarization, expression of cilia, apical versus basal secretion and apical versus basal virus entry [35]. To find out whether side-selective cell adhesion to BMs applies to different epithelial cells from different species, adhesion assays were also conducted with epithelial cells from the bovine corneal epithelium and the chick embryo retina, and endothelial cells from human dermal capillaries. Experiments with human fibroblasts were also performed to test for the adhesion of cells typical from stromal tissues. Dissociated cells were plated on top of the folded and flat-mounted human BMs, incubated for 15 minutes, and the BM flat mounts were washed to remove non-adherent cells. Results showed that MDCK cells, corneal epithelial, retinal cells and vascular endothelial cells adhered within 15 minutes of incubation firmly to the retinal/epithelial surface of all three BMs; the cells did not adhere to the vitreal/stromal side (Fig. 9A, B, D). By staining of the BM preparation for the 7S domain of collagen IV α3/4/5 to label the stromal surface, the side-specific adhesion of the cells was unequivocally confirmed (Fig. 9A–C). Cell adhesion assays with human fibroblasts showed that these cells adhered to both sides of the ILM at a similar rate (Fig. 9C, D). When retinal or dorsal root ganglia explants were cultured on folded BMs, axons grew out from the explants exclusively (retinal axons) or preferentially (DRG axons) on the retinal/epithelial side of all three BMs (n = 5 for each type of BM; Fig. 9E; Fig. S4A, B).

To test whether a contamination of vitreal hyaluronan or chondroitin sulfate proteoglycan on the ILM were responsible for the side-selective cell adhesion, the ILMs were pre-treated with chondroitinase or hyaluronidase and then tested for cell adhesion. Results showed that the enzyme treatment did not change the side-selective adhesion of MDCK cells to the retinal/epithelial side of the BMs (Fig. S5A, B; n = 5 for each enzyme).

**Figure 10:**
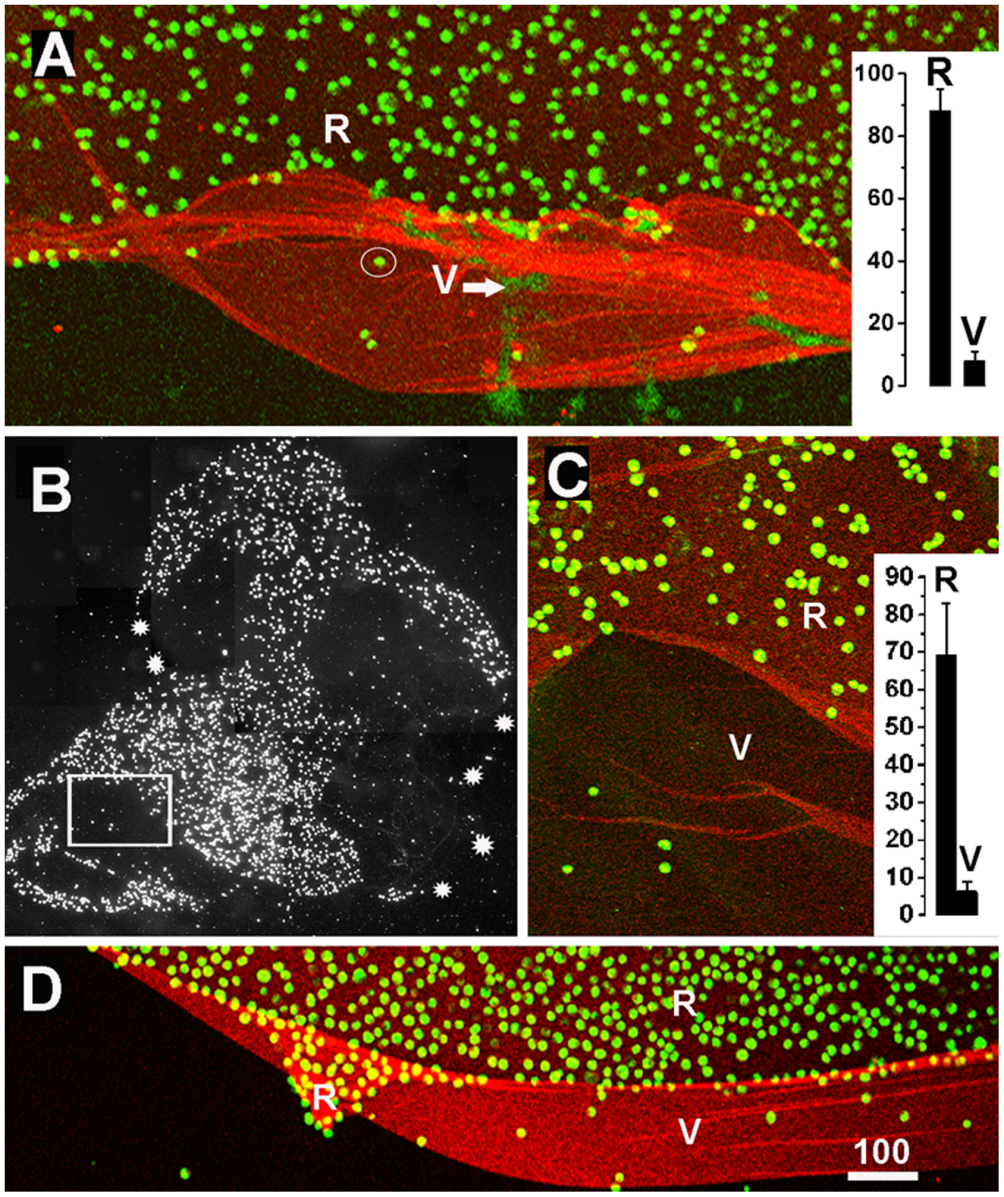
Cell adhesion assays showing the functional sidedness of mouse and embryonic chick ILMs. Mouse (A, two-week-old) and embryonic chick ILMs (B-D, embryonic day 9) were prepared and flat-mounted. When MDCK cells (green) were incubated on top of the ILM preparations, the cells prominently adhered to the retinal but not to the vitreal side of these BMs flat-mounts (A, D). The quantification of MDCK cell adhesion to either the retinal (R) or the vitreal (V) side of these BMs are shown in the inserted graphs (n = 10; A for mouse, C for chick). The white arrow in (A) points to DNA released from squashed retinal cells during the ILM flat-mount preparation. Its staining is very different from the crisp, circular DNA labeling of the adherent MDCK cells (circled). Chick ILMs were also flat-mounted by centrifugation (B, C). The dark field image of an entire chick BM preparation (B) shows that MDCK cells (white spots) adhered to only segments of the entire ILM (outlined by the white stars). The boxed area in (B) is shown at higher magnification in (C) confirming the selective adhesion of cells (green) to only one side of the ILM. The mouse ILM was stained for collagen IV (A), the chick ILMs were stained for laminin (C, D). Scale bar: A, C, D: 100 µm.

To find out whether a functional sidedness was also detectable in BMs from other species, two week-old and adult mouse ILMs were prepared by splitting whole-mounted retinas using poly-lysine-coated slides [25]. With this technique, the retinal side of the BM was consistently facing up while the vitreal surface was firmly attached to the slides. However, in about 20% of the cases segments of the ILMs were folded and the retinal and the vitreal surface were exposed side-by-side. Folding occurred at random sites of the ILM. MDCK and corneal epithelial cells were incubated as described and the adherent cells detected. Cell adhesion assays with these BMs showed that MDCK and corneal epithelial cells adhered only to the retinal and not to the vitreal side of the mouse ILMs (Fig. 10A; n = 10).

To test whether a selective adhesion can also be detected in newly assembled, embryonic BMs, ILMs were isolated from retinas of E9 chick embryos [24]. Free-floating ILMs were immobilized onto slides by centrifugations with random exposure of the vitreal or retinal side. Cell adhesion assays showed that MDCK and corneal epithelial cells adhered to restricted segments of the ILM, but did not adhere to other, often adjacent segments indicating an adhesion preference for one of the two sides of the BMs (Fig. 10B, C; n = 10). The chick ILMs were also prepared by splitting retinal whole mounts [25]. As for mouse ILM, in 20% of the cases segments of the embryonic chick ILMs were folded to expose the vitreal and retinal side side-by-side. Using these preparations in cell adhesion assays, it was found that adhesion of the MDCK and corneal epithelial cells was restricted to the retinal side of the BM, whereas few cell adhered to the vitreal side (Fig. 10D; n = 10).

## Discussion

The present experiments show that three human BMs have a sidedness that is detectable by an asymmetric protein/peptide epitope distribution, by differences in the biomechanical quality of both BM sides and by side-selective cell adhesion. The use of adult human BMs was critical for the initial phase of the project, since adult human BMs are thicker and easier to handle than BMs from short-lived animals, such as mice and rats. All tested human BMs roll up in a side-specific pattern and could, therefore, be mounted with either the epithelial or the stromal side up to test for side-specific properties. The same mounting technique was not as easily applicable for the much more delicate mouse or embryonic chick BMs.

### Asymmetric Distribution of BM Proteins

All tested human BMs are asymmetrically organized whereby the epithelial side is most easily distinguished by its abundance of laminin. The side-specific distribution of laminin was established using multiple anti-laminin antibodies, including a peptide-specific monoclonal antibody to laminin α5. Laminin 521 is the dominant family member in human ILM, LC and DM. Consistent with our data, a recent study on the human epidermal BM also detected an abundance of laminin or its epithelial surface [36]. We therefore, propose that the abundance of laminin is a characteristic for the epithelial surface of human BMs and may serve as a universal marker for the epithelial side of BMs.

Collagen IV α3/4/5 is the dominant collagen IV family member in the adult human ILM (Fig. 4B–E) and abundantly present in DM and LC, consistent with proteomic data [32]. Staining of the ILM and DM for the NC1 and the 7S domains of collagen IV α3/4/3 showed an N and C-terminal domain-specific labeling on opposite sides of the ILM and DM. It is tempting to speculate that collagen IV is organized such that the protein spans the entire width of the BMs with C-terminus on one side and the N-terminus on the opposite side. However, the adult human ILM and DM with several microns thickness are too wide for the 400 nm long collagen IVs. Further, antibody staining of the LC and DM leaves the central portion of the BMs less well stained suggesting antigen masking and, finally, an asymmetrical distribution of the NC1 and 7S domain of collagen IV was not detectable in the LC. The exact orientation of individual collagen IVs in BMs needs, therefore, further investigation. Nevertheless, the non-overlapping distribution of the C and N-terminal domains of collagen IV in two out of three human BMs questions the horizontal positioning of collagen IV as proposed in the current BM model. Whether the other members of the collagen IV family are similarly organized as the α3/4/5 chains is currently unknown, since 7S-specific antibodies for the α1/α2 chains were not available.

An asymmetric distribution of laminin and collagen IV was not detectable for the mouse and embryonic chick ILM; this could be due to technical problems to a) optically resolve the antibody labeling in the much thinner mouse and chick BMs and b) the lack of suitable antibodies; it is of note that domain- and (or) chain-specific antibodies are not available for mouse or chick collagen IV. However, it is also possible that the asymmetric protein distribution that we detected in adult human BMs is not present in these less than 100 nm-thin BMs.

A possible explanation for formation of the asymmetric structure of the human BMs could be the different origin of BM constituents: the laminins being contributed by the epithelium and the collagen IV by the stromal cells. However, distinct sites of synthesis as an explanation for the layered structure is unlikely for the ILM, since laminin and collagens IV are synthesized by the lens and ciliary body and both presented for ILM assembly via the vitreous [37]–[41]. Yet, it is also possible that the adult retina synthesizes laminin at quantities below the current detection limit, and that laminin accumulates as a distinct layer only after decades of human life. In fact, laminin synthesis has been documented in the adult mouse and adult human retina [31], [42]. The asymmetric structure of the BMs may also be explained by the way BMs are assembled, namely that a laminin core assembles first by binding to cellular receptors on the epithelial cell surfaces and that collagen IVs piles up on top of the laminin template as proposed earlier [1]. Further, a layered structure of BMs may also originate from the different rates of synthesis of BM proteins: laminin and collagen IV synthesis for ILM assembly is highest in embryonic stages and declines postnatally to a very low level [43]. It is conceivable that collagen IV synthesis in adult human eyes occurs at a slightly higher rate than any of the other BM proteins and, thus, piles up, tree ring-like, onto the earlier established laminin-dominated core. Consistent with this proposition is the fact that an asymmetric distribution of BM proteins is readily detectable in BMs from long-lived humans and undetectable in short-lived mouse and de-novo assembled ILM from chick embryos. Further, a detailed developmental study of the human DM showed two-phased growth of the DM: a fast fetal growth, and a slow postnatal growth resulting in a two-layered BM with a striated and an amorphous layer [44].

It is of note that ILMs, the DM and LC from fetal human eyes are indistinguishable from mouse and chick ILMs, vascular and muscle BMs: during fetal stages, they all share the same, typical textbook BM morphology with a distinct lamina densa and a thickness of 100 nm [20. 44. 45]. Postnatally, human ILMs, DMs and LCs increase in thickness and obtain the adult ultrastructure as shown in Fig. 1. BMs from mouse or rats never gain the thickness of adult human BMs because of their much shorter lifetime. An age-dependent increase in thickness has also been reported for the human epidermal BM [46] and in rat glomerular BMs [47] suggesting that age-dependent thickening is a normal process that affects many human BMs.

Uneven distributions of proteins within BMs have been reported earlier. A prominent labeling of the epithelial side for laminin has been documented for LC and DM [45], [48]. Further, a multi-layered structure of BMs is also suggested by the pathology of blistering diseases in humans: in the Herlitz-type junctional epidermolysis bullosa, the epidermal BM splits within the epidermal BM due to a mutation of laminin 332 [49]–[51]. Biochemical studies recently confirmed that the epidermal BM has a layered structure, with an abundance of laminin on the epithelial side [36]. However, in all previous studies, the asymmetrical distribution of proteins had always been considered a unique feature for the epidermal BM, the LC or the DM and have not been discussed in terms of a universal bi-functional organization that applies to all BMs. Further, side-specific biomechanical testing and cell adhesion assays for multiple BMs have not been published prior to this report.

### The Epithelial and Stromal Sides of BMs Differ in their Biomechanical Properties

AFM probing showed that the stromal side of all tested BMs is softer than the epithelial side. In addition, AFM measurements at different salt concentration showed that the two BM sides react differently, whereby the stiffness of the epithelial side more than doubles at high salt concentration, whereas the stiffness of the stromal sides remains unaffected. We propose that the difference in the biomechanical properties is due to differences in the protein composition and (or) a different degree of protein packaging at each of the BM sides. The latter proposition is based on the evaluation of TEM images of human ILMs in situ showing a high density of protein packaging at the retinal side and a loser packaging at the vitreal side [28]. We propose that the tighter packaging at the epithelial side results from a higher protein compression. The greater protein compression may have originated from the binding of a greater number of BM proteins by the abundant ECM-binding receptors of the adjacent epithelial cells as compared to the few adjacent cells and their receptors in the cell-sparse stromal tissue. We propose that when the BMs are isolated and no longer constrained by the adjacent epithelial layers, the proteins at the epithelial side of the BMs expand leading to the side-specific rolling that we observed for all human BMs.

### The Epithelial and Stromal Sides of BMs Differ in their Capacity to Promote Cell Adhesion

While an asymmetric structure was not detected in mouse and chick BMs, a functional sidedness was detectable for all BMs that were tested: epithelial cells, neuronal cells and growing axons preferentially adhered to the epithelial side of BMs, regardless of age, species and developmental stage. Earlier studies with crossections of human amnion BMs also showed a preference of axons to grow on the epithelial side of this BM [52]. The side-specific adhesion is certainly related to the expression of integrins and/or dystroglycan as the main cellular ECM receptors of the test cells. Dystroglycan, a major laminin-binding receptor, has been detected in MDCK [53] but not in corneal epithelial cells [54]. Since both cell types show the same side-specific adhesion, this protein is unlikely to be involved in side-selective cell adhesion to BMs. Similarly, the presence of absence of integrins does not provide a simple explanation for side-specific cell adhesion to BMs, since all epithelial cells used in our assays express laminin and collagen IV-binding integrins, including α2ß1, α3ß1 and α6ß4 [55]–[58]. It is still possible that the critical cell-binding sites are not exposed on the stromal BM surface, and, therefore, cells may not be able to adhere to this side. The current data show that the epithelial side of BMs is particularly well equipped to promote epithelial cell adhesion due to the high abundance of laminin, whereas the main function of the stromal side is to bind to connective tissue proteins, such as members of the fibrillar collagens. The data also suggest that the stromal surface of BMs has an anti-adhesive quality for epithelial cells that fibroblasts are able to overcome.

The conservation of BMs as a structure throughout all metazoans and the fact that BMs are the evolutionary oldest ECM proteins suggest an essential function of BMs for metazoan life [59]. We propose that the functional sidedness of BMs is universal for all metazoans BMs, consistent with countless light and electron microscopy images of BMs zones showing a cell-rich epithelial layer on one side of BMs and a cell-sparse and collagen-1-rich, connective tissue layer on the other side. Due to the bi-functional nature, we hypothesize that BMs participate in organizing multicellular organisms in alternating layers of epithelial and connective tissues. In this role, BMs not only serve to connect different types of tissues, but also to prevent the formation of directly apposed epithelial cell layers. The alternating organization of epithelial and connective tissues is likely to be essential for the existence of all complex multicellular organisms, since epithelial cell layers are mechanically weak and a-vascular, whereas connective tissue layers are mechanically strong and carry the vascular and nerve supply. The alteration of both types of tissues provides an optimum of epithelial function with the necessary mechanical support from the adjacent connective tissue.

Exceptions to this rule are the glomerular BMs in the kidney, the alveolar BMs in the lung and Bruch’s membrane that have epithelial layers on both sides. However, these BMs are formed by the fusion of an epithelial and an endothelial BM after losing the intersecting connective tissue layers. It is worth noting that both apposing epithelia in lung and kidney are highly specialized for optimal filtration or oxygen transfer and a stiff mechanical support of these structures is less important. A true exception of a BM with two adjacent epithelial-like cell layers is the capillary BMs in the brain, retina and spinal cord with the glial and the endothelial cell layers on either side. We propose that in this case mechanical stability was sacrificed for a high density of neuronal cells and synapses.

## Supporting Information

Figure S1 Stiffness of ILMs in hypotonic (black), isotonic (blue) and hypertonic (red) salt concentrations as measured by AFM. Typical stiffness measurements of a vitreal ILM surface at different salt concentrations are shown in panel (A); the stiffness data for the vitreal surface are very similar for all salt concentrations. In contrast, the stiffness of the retinal side from two different ILMs is more than doubled in hypertonic buffer as compared to the stiffness measured in hypotonic and isotonic buffer (B, C). The average stiffness is indicated on top of each of the data distribution curves.
(TIF)

Figure S2 **Control staining of a folded human ILM with an antibody to fibronectin (red).** Fibronectin is not present in normal ILMs. The absence of labeling shows that there is no non-specific staining of the BMs with secondary antibodies or non-relevant antibodies (A). Evidence that an ILM was present in this sample was provided by staining of the BM with an antibody to 7S collagen IV α3 (green; B) that prominently stains the vitreal (V) and much less the retinal (R) side of the ILM. Bar: 100 µm.
(TIF)

Figure S3 **Asymmetric distribution of laminin and collagen IV 7S in human ILMs that were obtained by ILM peeling**. Both ILM samples are rolled up (A). When flat-mounted onto glass slides and stained with an antibody to the 7S domain of collagen IV α3/4/5 the vitreal surface is strongly labeled (green; B, C). Additional labeling for laminin (red) shows the prominence of laminin on the retinal side of the ILM (C). Scale Bar: 100 µm.
(TIF)

Figure S4 **Preferred outgrowth of axons from chick dorsal root ganglia on the retina side (R) of human ILMs.** When dorsal root ganlia were placed in folded ILMs, axons outgrowth on the retinal side of the BM (R) was profuse, fast and resulted in long, defasciculated axons after 24 hours of incubation (A). Axons on the vitreal side of the ILM (V) were very short, greatly reduced in number, and highly fasciculated (A, B). Panel B shows the area marked by an arrow in A at higher power. Bars: A: 200 µm; B: 50 µm.
(TIF)

Figure S5 **Side-selective adhesion of cells to ILM flat mounts after hyaloronidase or chondroitinase pre-treatment.** The ILMs were been flat-mounted on slides and treated with 1 mg/ml hyaluronidase (Sigma; A) or 250 mU/ml chondroitinase ABC (Seikagaku; B) in PBS/1 mg/ml BSA for 6 hours. The BMs were washed and MDCK cells were plated and incubated on top of the BM substrates for 15 minutes. The cells were washed off; the samples were fixed and stained for 7S collagen IV (red) and for cell nuclei with Sytox Green. The two panels showed that the side-selective cell adhesion of MDCK cells was not affected by the treatment with both enzymes, indicating that the inhibitory property of the vitreal side of the ILM is not due to residual chondroitin sulfate proteoglycans from the former adjacent vitreous.
(TIF)

Table S1 **Overview of basic eye donor characteristics.**
(DOCX)
