# Supplementary figures and images for: The Bi-Functional Organization of Human Basement Membranes
Source: PLoS One. 2013 Jul 3;8(7):e67660. doi: 10.1371/journal.pone.0067660 (PMC3700973; doi:10.1371/journal.pone.0067660)

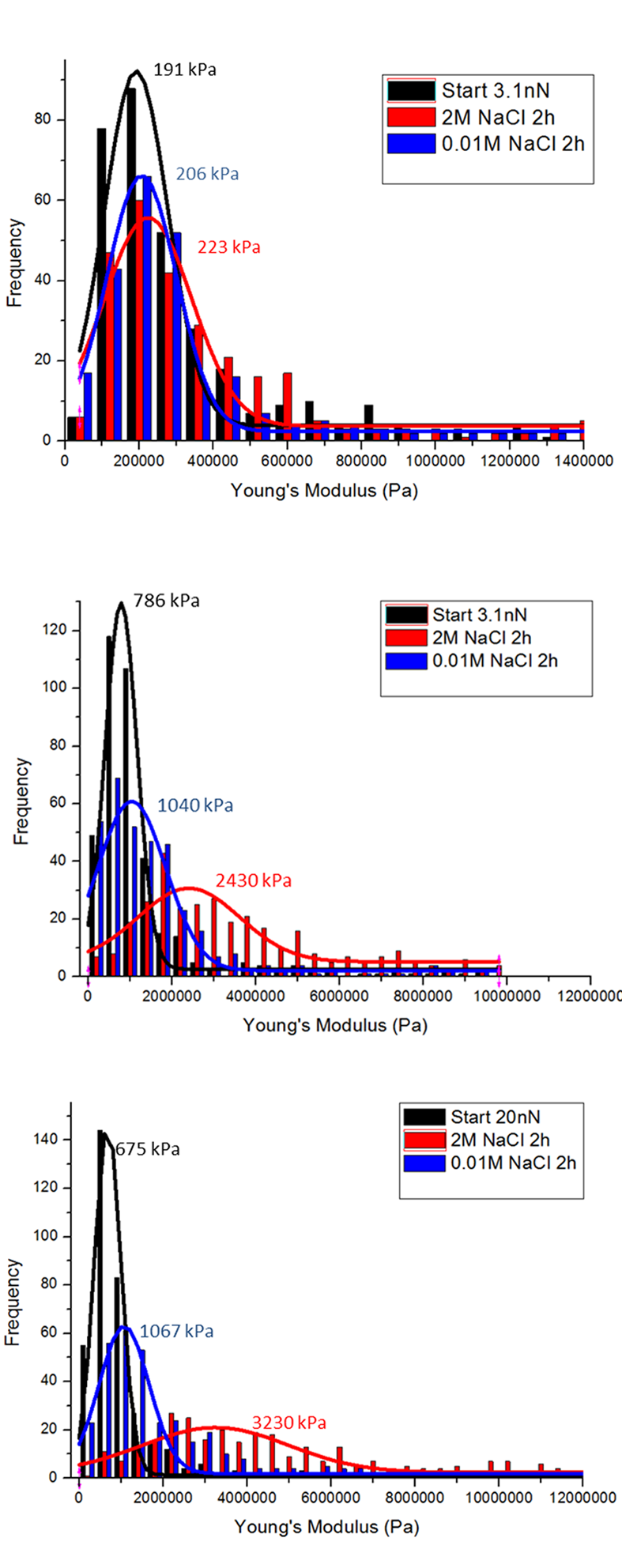

Supplement: Figure S1 — Stiffness of ILMs in hypotonic (black), isotonic (blue) and hypertonic (red) salt concentrations as measured by AFM. Typical stiffness measurements of a vitreal ILM surface at different salt concentrations are shown in panel (A); the stiffness data for the vitreal surface are very similar for all salt concentrations. In contrast, the stiffness of the retinal side from two different ILMs is more than doubled in hypertonic buffer as compared to the stiffness measured in hypotonic and isotonic buffer (B, C). The average stiffness is indicated on top of each of the data distribution curves. (TIF) [file pone.0067660.s001.tif]

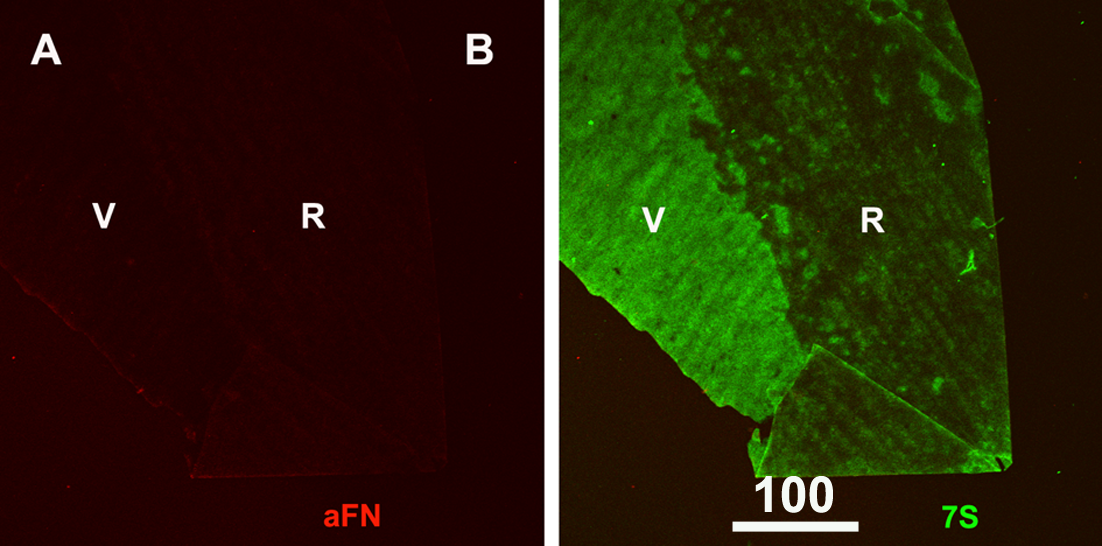

Supplement: Figure S2 — Control staining of a folded human ILM with an antibody to fibronectin (red). Fibronectin is not present in normal ILMs. The absence of labeling shows that there is no non-specific staining of the BMs with secondary antibodies or non-relevant antibodies (A). Evidence that an ILM was present in this sample was provided by staining of the BM with an antibody to 7S collagen IV α3 (green; B) that prominently stains the vitreal (V) and much less the retinal (R) side of the ILM. Bar: 100 µm. (TIF) [file pone.0067660.s002.tif]

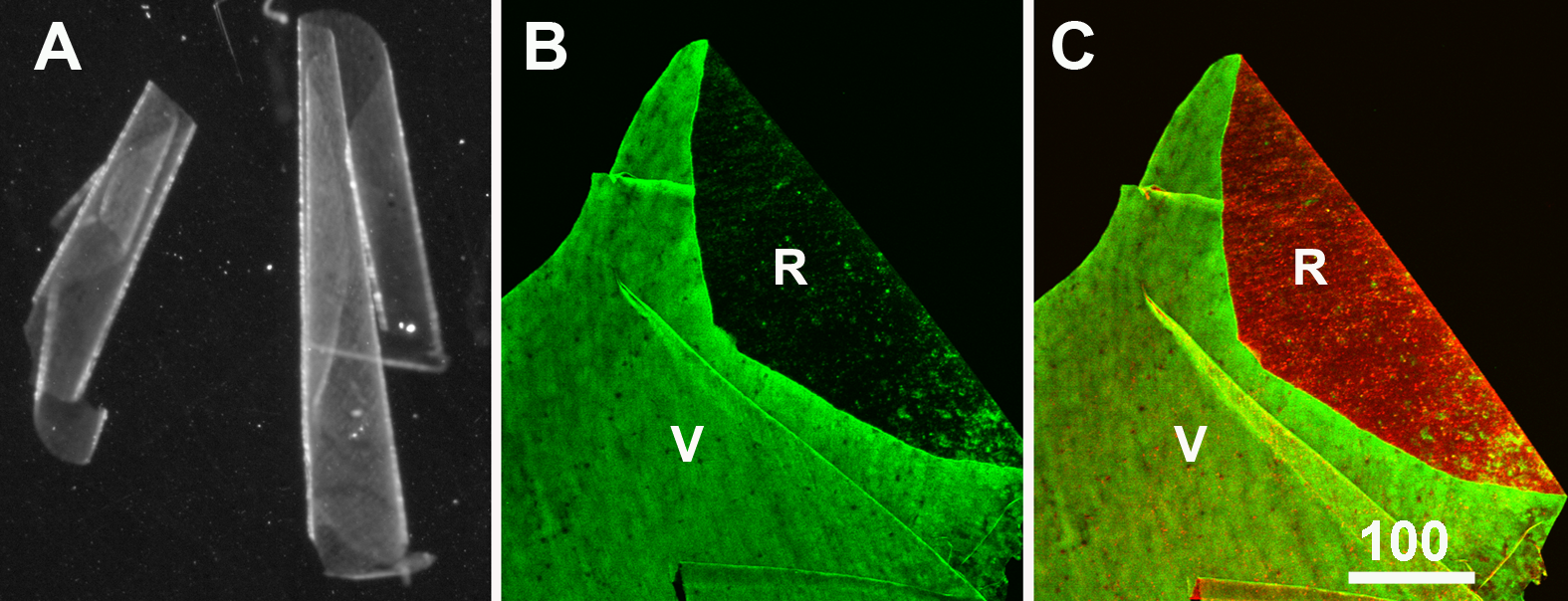

Supplement: Figure S3 — Asymmetric distribution of laminin and collagen IV 7S in human ILMs that were obtained by ILM peeling. Both ILM samples are rolled up (A). When flat-mounted onto glass slides and stained with an antibody to the 7S domain of collagen IV α3/4/5 the vitreal surface is strongly labeled (green; B, C). Additional labeling for laminin (red) shows the prominence of laminin on the retinal side of the ILM (C). Scale Bar: 100 µm. (TIF) [file pone.0067660.s003.tif]

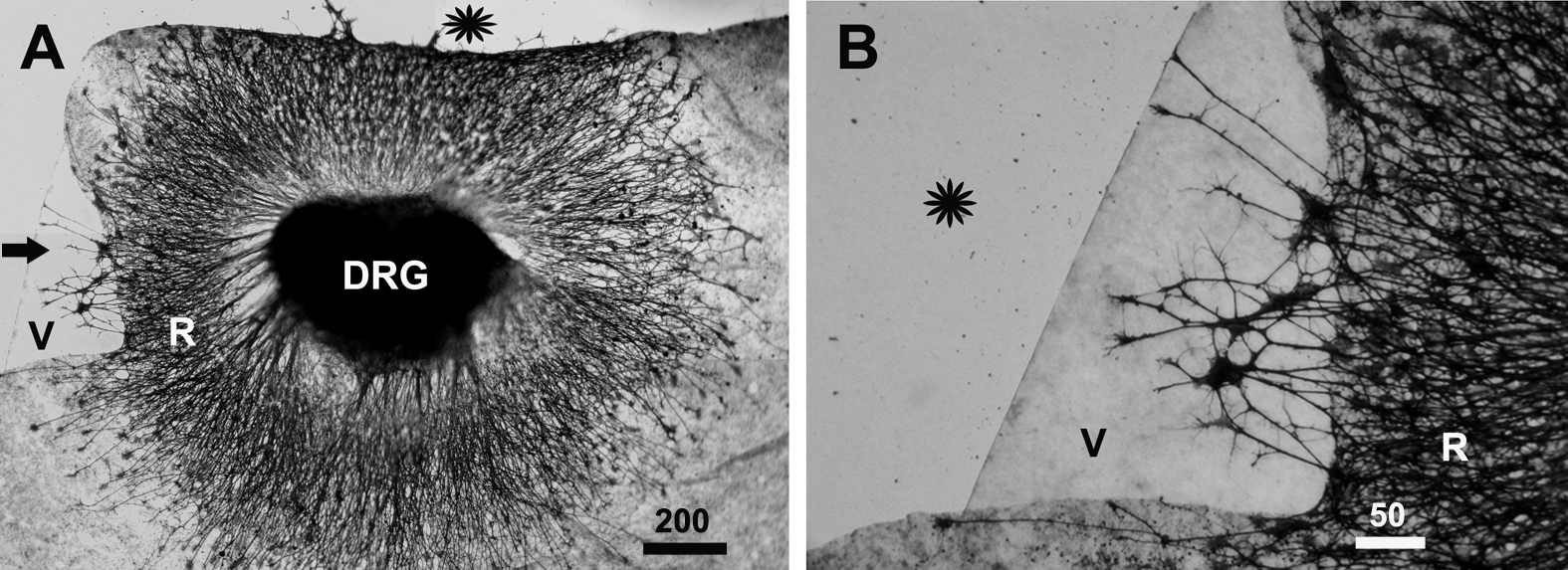

Supplement: Figure S4 — Preferred outgrowth of axons from chick dorsal root ganglia on the retina side (R) of human ILMs. When dorsal root ganlia were placed in folded ILMs, axons outgrowth on the retinal side of the BM (R) was profuse, fast and resulted in long, defasciculated axons after 24 hours of incubation (A). Axons on the vitreal side of the ILM (V) were very short, greatly reduced in number, and highly fasciculated (A, B). Panel B shows the area marked by an arrow in A at higher power. Bars: A: 200 µm; B: 50 µm. (TIF) [file pone.0067660.s004.tif]

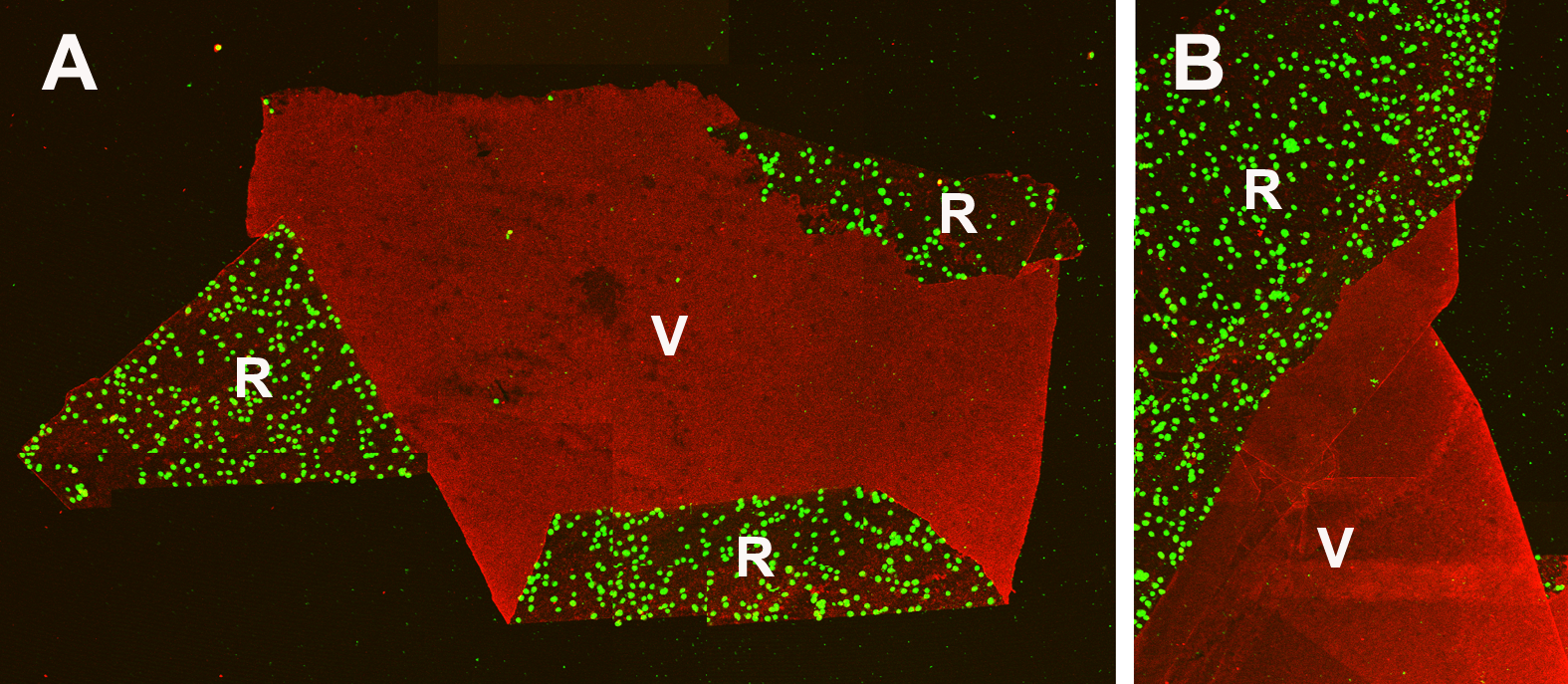

Supplement: Figure S5 — Side-selective adhesion of cells to ILM flat mounts after hyaloronidase or chondroitinase pre-treatment. The ILMs were been flat-mounted on slides and treated with 1 mg/ml hyaluronidase (Sigma; A) or 250 mU/ml chondroitinase ABC (Seikagaku; B) in PBS/1 mg/ml BSA for 6 hours. The BMs were washed and MDCK cells were plated and incubated on top of the BM substrates for 15 minutes. The cells were washed off; the samples were fixed and stained for 7S collagen IV (red) and for cell nuclei with Sytox Green. The two panels showed that the side-selective cell adhesion of MDCK cells was not affected by the treatment with both enzymes, indicating that the inhibitory property of the vitreal side of the ILM is not due to residual chondroitin sulfate proteoglycans from the former adjacent vitreous. (TIF) [file pone.0067660.s005.tif]
