# Supplementary material for: The Bi-Functional Organization of Human Basement Membranes
Source: PLoS One. 2013 Jul 3;8(7):e67660. doi: 10.1371/journal.pone.0067660 (PMC3700973; doi:10.1371/journal.pone.0067660)
Supplement: Table S1 — Overview of basic eye donor characteristics. (DOCX) [file pone.0067660.s006.docx]

| **#** | **age** | **gender** | **race** | **Cause of death** | **Med history** |
| --- | --- | --- | --- | --- | --- |
| **1** | **19** | **m** | **b** | **MVA** | **None** |
| **2** | **45** | **m** | **w** | **Accident** | **Broken ankle** |
| **3** | **62** | **m** | **w** | **MVA** | **HTNx10 years** |
| **4** | **66** | **m** | **w** | **CA** | **Chemo; CVA 10 years ago** |
| **5** | **80** | **m** | **w** | **AAA** | **None** |
| **6** | **56** | **m** | **w** | **MI** | **None** |
| **7** | **63** | **m** | **w** | **MI** | **Pneumonia, asbestosis** |
| **8** | **72** | **m** | **w** | **MI** | **HTN, PVD** |
| **9** | **46** | **f** | **w** | **Cardiac arrest** | **HTN, Crohn Disease** |
| **10** | **83** | **m** | **w** | **CVA** | **HTN, PVD, Defibrillator** |
| **11** | **80** | **m** | **w** | **MI** | **HTN, GERD, Pacemaker** |
| **12** | **82** | **m** | **w** | **MI** | **HTN, COPD** |
| **13** | **72** | **f** | **w** | **ICH** | **COPD, HTN** |
| **14** | **66** | **m** | **w** | **ICH** | **Dementia** |
| **15** | **47** | **m** | **w** | **Suicide** | **HTN** |
| **16** | **80** | **m** | **w** | **MI** | **Pacemaker, GERD** |
| **17** | **50** | **f** | **w** | **ICH** | **None** |
| **18** | **54** | **m** | **w** | **MI** | **HM** |
| **19** | **53** | **m** | **b** | **MI** | **Bipolar Disorder, Drug abuse** |
| **20** | **84** | **m** | **w** | **Accident** | **Pacemaker, COPD** |

Table 1: Overview of basic eye donor characteristics. COPD = Chronic obstructive pulmonary disease; CVA = Cerebrovascular accident; GERD = Gastroesophageal reflux disease; HM = Heart Murmur; HTN = Arterial hypertention; ICH = Intracerebral hemorrhage; MI = Myocardial infarction; PVD = Peripheral vascular disease; MVA = motor vehicle accident; CA = cancer. AAA = abdominal arterial aneurism;
